# Supplementary material for: A model of thalamo-cortical interaction for incremental binding in mental contour-tracing
Source: PLoS Comput Biol. 2025 May 8;21(5):e1012835. doi: 10.1371/journal.pcbi.1012835 (PMC12061125; doi:10.1371/journal.pcbi.1012835)
Supplement: S1 Text — Further details on methods regarding the derivation of the steady-state iteration employed in the simulation, as well as additional details on model parameters and stimulus creation. (PDF) [file pcbi.1012835.s001.pdf]

A model of thalamo-cortical interaction for incremental binding in mental contour-tracing Supporting information

Daniel Schmid[1*] and Heiko Neumann[1]

[1]Institute for Neural Information Processing, Ulm University, Ulm, Baden-Württemberg, Germany
[*]daniel-1.schmid@uni-ulm.de

March 10, 2025

S1 Further details on methods

S1.1 Derivation of steady-state iteration

The temporal evolution of neurons in the model's visual cortex and interfacing modules are described in terms of first-order differential equations (Eqs 9, 10, and 15). Together they define a system of dynamical equations as recapitulated below (see Sect S1.2 for details on parameters).

Generally, these equations can be described as

$$\dot{v}_i = F(\mathbf{v}, I, t)), \tag{A}$$

where the temporal change $\dot{v} = \frac{dv}{dt}$ of each system variable v_i is dependent via some function F on the current state of the system, comprised of the set of all state variables $\mathbf{v}$, external inputs I, and potentially the time t itself. For the presented model, no explicit dependency of the state evolution on time exists, and all presented simulations have been performed on static input, resulting in $F(\mathbf{v}(t), I)$.

The model is defined by the following equations (Eqs 9, 10, and 15):

$$\tau_b^V \dot{v}_b^V = -v_b^V \cdot g_b^{V,Leak} + (\beta_b^V - v_b^V) \cdot g_b^{V,Exc} - \kappa_b^V \cdot v_b^V \cdot h_b^{V,Inh}, \quad V \in \{V1, V2, V4\}\,, \tag{B}$$
$$\tau_a^V \dot{v}_a^V = -v_a^V \cdot g_a^{V,Leak} + (\beta_a^V - v_a^V) \cdot g_a^{V,FB}, \quad V \in \{V1, V2, V4\}\,. \tag{C}$$
$$\tau^{HO} \dot{v}^{HO} = -v^{HO} \cdot g^{HO,Leak} + \left(\beta^{HO} - v^{HO}\right) \cdot g^{HO,Exc}. \tag{D}$$

Wherever possible, the model differential equations were solved at equilibrium in response to a constant input stimulus in order to reduce processing time. For each of the three Eqs B, C, and D we obtain the equilibrium responses

$$v_{b,\infty}^{V} = \frac{\beta_b^V \cdot g_b^{V,Exc}}{v_b^V \cdot g_b^{V,Leak} + v_b^V \cdot g_b^{V,Exc} + \kappa_b^V \cdot v_b^V \cdot h_b^{V,Inh}}, \qquad V \in \{V1, V2, V4\}, \tag{E}$$

$$v_{a,\infty}^{V} = \frac{\beta_a^V \cdot g_a^{V,FB}}{v_a^V \cdot g_a^{V,Leak} + v_a^V \cdot g_a^{V,FB}}, \qquad V \in \{V1, V2, V4\}, \tag{F}$$

$$v_{\infty}^{HO} = \frac{\beta^{HO} \cdot g^{HO,Exc}}{v^{HO} \cdot g^{HO,Leak} + v^{HO} \cdot g^{HO,Exc}}. \tag{G}$$

There, dependencies on the system's state by some non-linear functions exist, e.g., H for $g_b^{V,Exc} = H(\mathbf{v})$. Similar to earlier work [1], we efficiently obtain a useful proxy of the system's dynamics by evaluating the equations iteratively and re-inserting the obtained new estimates of the equilibrium state $\mathbf{v}_\infty$. Overall, this yields the iteration rules in Eqs 18, 19, and 20.

S1.2 Additional implementational details

The listed parameters were determined by manual search (Table A). While the parameter choice proved itself robust for the given experiments, no dedicated robustness tests have been performed. Yet, during optimizing for these experiments, the parameter regime was graceful around the chosen values, so that the choice of integer numbers was often reasonably good enough. Conversely, this means that a computationally more demanding optimization of the parameters could yield even better quantitative fits to experimental data.

Some of the parameter choices turned out to be less critical than others. For parametrization of visual cortex model neuron leak and reversal potential parameters were uncritical and kept at default values, $g^{V,Leak}$ and β^V, respectively. Likewise, the number of orientation channels n_θ was rather uncritical, as long as it was high enough to sufficiently cover the input space.

Tuning of spatial model parameters proved to be important to achieve growth-cone-like variations in incremental binding speed (Sects 2.2.2 and 2.2.3). Establishing a hierarchical scale space by down-sampling in combination with inversely proportional kernel sizes yielded the best outcome. These kernel sizes were achieved by constant kernel parametrizations $\sigma^{V,C}$ for cortico-cortical projections in combination with the *resize* operation to express the proper scale relations between areas. For thalamo-cortical projections approximately up-scaling the spatial extent of the kernel ρ^V by the respective ratio together with the *resize* operation resulted in a nearly constant neighborhood in terms of the number of neurons per module area covered by the gating signal.

The most critical tuning of parameters was with respect to the relative contributions of excitation and inhibition within the model. For the contextual feedback input to have an effect on the cell's basal membrane potential the apical impact factor λ^V had to be in the order of magnitude of 10. To establish enough specificity in terms of the formed compatibilities between features via feedback projections it was important to up-scale the impact of inhibitory sub-fields via parameter ξ (Fig A). This way, incompatible features effectively can provide counterevidence and establish a veto against propagation at the respective scale, e.g., in case a parallel distractor line would fall into the inhibitory sub-field. The local excitation-inhibition balance

Table A: Parameters used throughout the different incremental grouping experiments.

**Parameter**	**Value**	**Explanation**
**V1-V4**		
n_θ	12	Number of orientation feature channels
$g^{V,Leak}$	1.0	Leak constant, $V \in \{V1, V2, V4\}$
β^V	1.0	Excitatory reversal potential, $V \in \{V1, V2, V4\}$
κ^V	7.5	Divisive inhibition factor, $V \in \{V1, V2, V4\}$
λ^V	15.0	Apical impact factor, $V \in \{V1, V2\}$
$k^{V1,FF}$	0.25	Re-scaling input activity
$k^{V,FF}$	3.0	Re-scaling activity between areas, $V \in \{V2, V4\}$
s^{V1}	1	Spatial down-scaling from input to area V1
s^V	1/2	Spatial down-scaling to subsequent areas, $V \in \{V2, V4\}$
s^{HO}	2/3	Spatial down-scaling from V1 to HO
**HO**		
$g^{HO,Leak}$	0.7	Leak constant
β^{HO}	1.0	Excitatory reversal potential
$k^{HO,S}$	8.0	Input scaling factor
$k^{HO,V1}$	16.0	Input scaling factor
$k^{HO,V2}$	4.0	Input scaling factor
$k^{HO,V4}$	1.0	Input scaling factor
**Kernels**		
$\sigma^{V,FF}$	2.0	envelope standard deviation, $V \in \{V1, V2, V4\}$
$\sigma^{V,Inh}$	1.0	kernel standard deviation, $V \in \{V1, V2, V4\}$
$\sigma^{V,FB}$	2.0	envelope standard deviation, $V \in \{V1, V2\}$
ξ	2.5	Inhibitory feedback component scaling factor
$\rho_x^{HO,V}$	3	Spatial extent of kernel, V to HO, $V \in \{V1, V2, V4\}$
$\rho_x^{V1,HO}$	5	Spatial extent of kernel, HO to V1
$\rho_x^{V2,HO}$	9	Spatial extent of kernel, HO to V2
$\rho_x^{V4,HO}$	13	Spatial extent of kernel, HO to V4

was further maintained by properly scaling excitatory input activity per area $k^{V,FF}$ and local divisive inhibition κ^V.

It was equally important to adjust the summed evidence against the non-linearity f^{HO} for neurons in the interfacing module. Balancing the leakage $g^{HO,Leak}$ and the summed excitatory input effectively adjusted the working range, such that only HO neurons that received input from up-modulated neurons in V1, or V1 and higher, or from the task module S. The relative scaling between V1 to V4 were chosen in accord to the spatial down-scaling factor, since a single V4 neuron can affect many more HO neurons at once than V1 neurons due to their different spatial scaling. Exchanging the simple summation of the input from the different areas (Eq 16) by a non-linear interaction scheme, e.g., a maximum selection among inputs, could make this working range more robust against the specific parameter choice. Yet, we omitted such countermeasure for the sake of a more simplistic model which was still able to capture the observed incremental binding correlates.

Beyond the model parametrization it was as well important to parametrize the input to the model in terms of spatial scale of the input structures, i.e., contours, and input strength. While model parameters were kept constant for all conducted stimulations, input parameters were adjusted between experiments. If the spatial scale would mismatch the kernel sizes $k^{V1,FF}$ the model would fail to represent the structures properly. If the input strength would be too small, then it would be insufficient to drive the model's feedforward representation. Conversely, if the input strength would be too large, neural activities would lie outside the working regime and HO neurons would treat arbitrary input locations as up-modulated input signals opening gates at too many locations. This indicates that the currently employed inhibitory local circuit is designed to establish *competition* between neurons of different feature tuning, i.e., orientation selectivity, but not to establish *normalization* among input ranges. Such normalization would be required to extend the model to become invariant against fluctuations in local input intensity and scale.

The presented model describes a fully convolutional architecture (Sect 4). Each spatial and feature interaction is described via local neighborhood relations that can be computed by applying the convolution operator onto the respective representation and filter kernel. The filters have been described in Sect 4.1.1 and a visualization of relative filter sizes and exemplary filters is provided in Fig 12. The feedback filters constitute the most complex of the connectivity schemes and the complete filter bank between model area V2 and V1 can be seen in Fig A.

S1.3 Stimulus parameterization and creation

Since the model lends itself to fully convolutional processing, the number of neurons in each area does not need to be prespecified. In fact, the only parameters that need to be prespecified per model area are the number of feature channels, i.e., specific orientations to represent, and the relative size, i.e., spatial resolution, of each area (cf. Table A rescaling factors s^V). Thus, the spatial extent of each model area in absolute numbers is directly determined by the input's spatial extent (Table B).

Panel creation has been established in two ways, a pixel-wise specification for tightly controlled stimulus panels, and an interpolation-based method from function values for more complex configurations. The pixel-wise approach has been applied to stimuli used in the experiments with varying target-distractor distances (Sects 2.2.1,

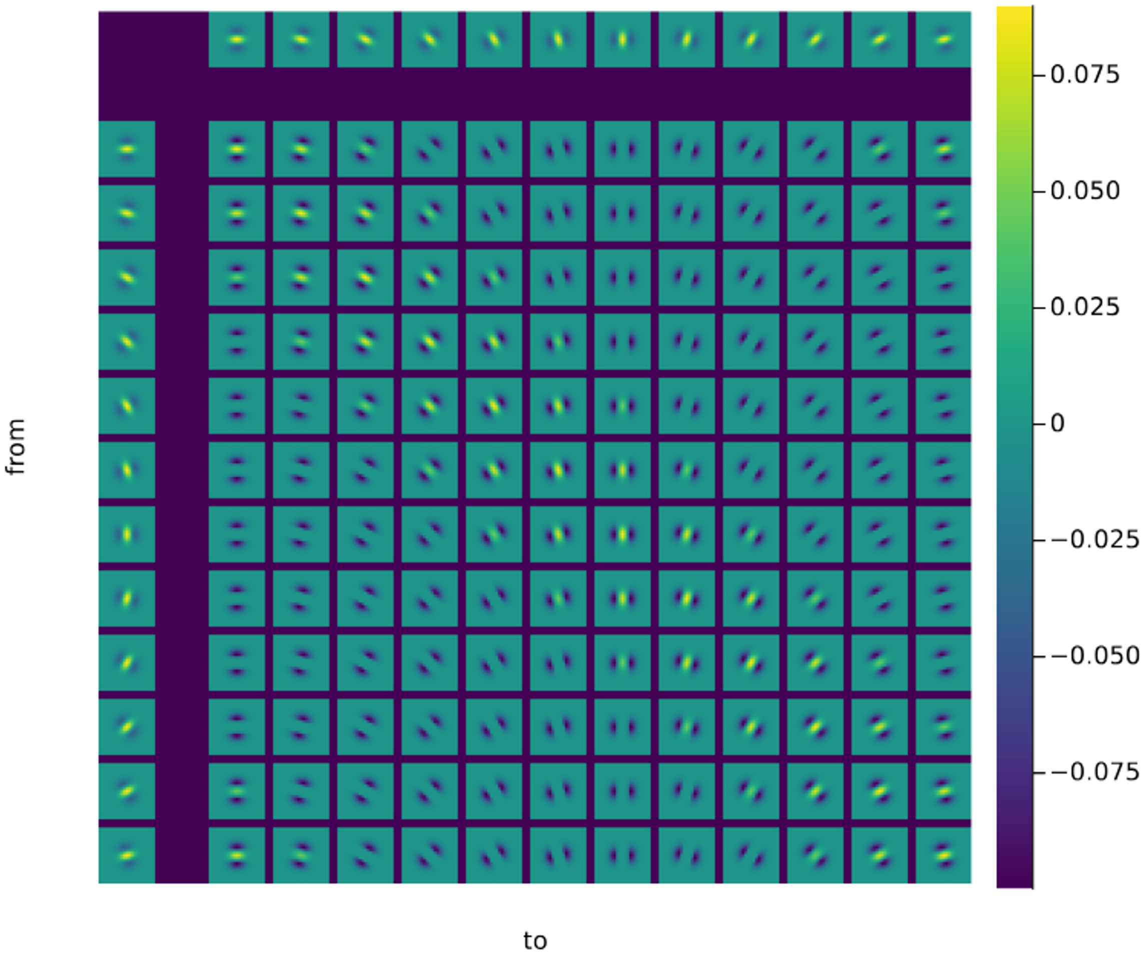

Fig A. Spatial weights of feedback couplings between filters of different orientations.
Depicted are feedback kernels from V2 to V1 neurons for all combinations of V2 and V1 orientation selectivity (first column and top row depict the respective selectivity). Phase and orientation of feedback kernels is determined based on the V1 neuron's preferred phase and orientation. Excitatory strength depends on whether the V2 neuron's selectivity matches the V1 neuron's one and is maximal for the same tuning and purely inhibitory for orthogonal tunings. This display extends the display in Fig 12D showing all possible connection patterns.

Table B: Stimulus parameters for main paper experiments.

**Parameter**	**Value**	**Explanation**
**Varying distance**		Varying target-distractor distance stimuli (Sects 2.2.1, 2.2.2, and 2.2.3)
values	$[0.0, 1.0]$	normalized value range
size(x,y)	96×312	spatial extent (horizontal and vertical)
**Crossing**		Crossing stimuli (Sect 2.2.4)
values	$[0.0, 1.0]$	normalized value range
size(x,y)	126×186	spatial extent (horizontal and vertical)
**Complex stimuli**		Set of different stimuli (Sect 2.2.5, S2 Text)
values	$[0.0, 0.5]$	normalized value range
**Lines**		`Lines` stimuli
size(x,y)	177×177	spatial extent (horizontal and vertical)
**Dashed**		`Dashed` lines stimuli
size(x,y)	177×177	spatial extent (horizontal and vertical)
**Rings**		`Rings` stimuli w/ distractor rings
size(x,y)	209×209	spatial extent (horizontal and vertical)
**Single rings**		`SingleRings` stimuli w/o distractor rings
size(x,y)	209×209	spatial extent (horizontal and vertical)
**Spiral**		`Spiral` stimulus
size(x,y)	231×231	spatial extent (horizontal and vertical)
**Pathfinder14**		`Pathfinder14` (Sect 2.2.5, S2 Text)
values	$[0.0, 0.65]$	normalized value range
size(x,y)	200×200	spatial extent (horizontal and vertical)

2.2.2, and 2.2.3) and crossings (Sect 2.2.4). The interpolation-based method has been applied to stimuli used in experiments with a diverse set of more complex stimuli (Sect 2.2.5, S2 Text). Also, the additional experiments reported in the supporting information have been based on the interpolation-based method (see S2 Text).

For the experiments with pixel-wise specified stimuli the input data r^{Inp} (cf. Eq 1) has been created in two steps. First, a contour of respective shape has been defined on a pixel grid with a line width of 1 pixel and a grayscale intensity value of 1. To have a smoother transition for diagonal elements two neighboring pixels have been set to intensity values of 0.75 instead of setting one pixel in an 8-neighborhood to 1. Second, the resulting image has been padded by 8 pixels to each side, blurred using Gaussian filtering ($\sigma = 1.0$) and subsequently up-scaled by a factor of 3 to the desired input size followed by linear interpolation. All input images have been normalized to a maximum of 1 (Table B). For the case of varying distances between target and distractor curve, the wide distance segment had a spacing of 8 empty pixels, and the narrow distance segment had a spacing of 4 pixels. The vertical extent of the lines was 81 pixels. The proportion of narrow vs. wide segment length has been varied parametrically between 0.15 and 0.85, which provided a range in which the stimulus properties stood intact, and a constant overall curve length was maintained. The extent of the narrow segment has been positioned symmetrically around the contour's longitudinal center.

For the crossing stimuli the same procedure has been applied. The spacing between the two lines in the defined pixel grid was 11 pixels and the straight line 42 pixels long.

The attentional seed values have been provided via a separate map $g^{HO,S}$ (cf. Eq 16). There, the creation procedure consisted of pixel-precise definition, no blurring, and up-scaling. Each seed of attention has been provided as a single pixel of intensity value 1 at the corresponding end point of the respective contour.

For the set of complex stimuli (all but `Pathfinder14`) a three-step procedure created input images from mathematically specified contours. First, sampling of from a function evaluation yielded values and sample positions in continuous Cartesian coordinates. The evaluated function depended on the exact stimulus, e.g., a line equation for the `lines` stimuli, or an arc equation for the `rings` stimuli. Additional sampling of background values has been provided from the same respective function, e.g., two lines in parallel to the stimulus line. These background samples were offset towards the respective direction from the stimulus structure by half the structure's specified line width parameter. For the complex stimuli, the line width was specified as 1 pixel resulting in directed offset values of 0.5 pixels. The respective functions were called with respective parametrizations to, e.g., establish a set of three parallel lines (`lines15` and `lines40` cases). During a second step, an image has been created by interpolation of values onto the image's pixel grid. For the interpolation Gaussian functions centered at each pixel position have been evaluated based on the set of sample coordinates and values from the function evaluation. Gaussians had a standard deviation of $\sigma^p = 0.5$ pixels. During the third and last step, interpolated image values were normalized to a defined value range. Therefore, small image values from long-tail Gaussian interpolated were truncated by setting all values smaller or equal to 0.15 to zero, and then the value range was normalized to an interval between a specified minimum and maximum (cf. Table B).

For the shown model performance on `Pathfinder14` a different preprocessing procedure has been performed. First, stimulus input size has been resized to 200×200. Then, all values bigger or equal to 0.15 have been set to 1.0 again to remove most inter-

polation artifacts. Finally, the value range has been rescaled to $[0, 0.65]$ (cf. Table B). The `Pathfinder14` stimulus corresponds to the pathfinder display versions with one end point [2].

References

[1] Bayerl P, Neumann H. Disambiguating Visual Motion Through Contextual Feedback Modulation. Neural Computation. 2004;16(10):2041–2066. doi:10.1162/0899766041732404.

[2] Linsley D, Karkada Ashok A, Govindarajan LN, Liu R, Serre T. Stable and expressive recurrent vision models. In: Larochelle H, Ranzato M, Hadsell R, Balcan MF, Lin H, editors. Advances in Neural Information Processing Systems. vol. 33. Curran Associates, Inc.; 2020. p. 10456–10467.
