# Supplementary material for: A model of thalamo-cortical interaction for incremental binding in mental contour-tracing
Source: PLoS Comput Biol. 2025 May 8;21(5):e1012835. doi: 10.1371/journal.pcbi.1012835 (PMC12061125; doi:10.1371/journal.pcbi.1012835)
Supplement: S2 Text — Extension of presented results and additional simulations on a broader range of stimuli. (PDF) [file pcbi.1012835.s002.pdf]

# A model of thalamo-cortical interaction for incremental binding in mental contour-tracing Supporting information

Daniel Schmid[1*] and Heiko Neumann[1]

[1]Institute for Neural Information Processing, Ulm University, Ulm, Baden-Württemberg, Germany
[*]daniel-1.schmid@uni-ulm.de

March 10, 2025

## S2 Results on additional stimuli

The model has been tested on a broader range of stimuli in addition to the line stimuli on which the model's growth cone properties were investigated and the computational properties on simple contour crossings has been analyzed (Sect 2.2). On the one hand, these additional simulations showcase how well the model can generalize based on a simplistic representation only consisting of oriented contrast selective neurons. On the other hand, the same stimuli but with different parameters show the model's limitations and provide insights into why and how the base representation needs to be extended beyond oriented contrast selectivity.

### S2.1 Additional details on results from complex stimuli

For the complex stimuli (Sect 2.2.5) further results are depicted and briefly discussed here. The `spiral` stimulus shows a trend from smaller to larger incremental binding speed with increasing radii. It plateaus at around the speed of the `rings` cases (Fig A) constituting a special case to consider when interpreting the aggregate data (Fig 9C). For the other stimuli, the variation in speed did not follow a similar trend but was rather independent of the probe's distance along the curve from the attentional seed point (Fig A). Therefore, a curvature-dependent decrease in the model's tracing speed is observed only for large curvatures (small distances from the starting point in the `spiral` case; smaller overall speed for the `singleRing15` compared to the other `singleRing` cases).

### S2.2 Additional experiments with intersecting stimuli

The intersection stimulus yields insights into the dynamical interaction between the base representation (as initial interaction skeleton), and the incremental attentional

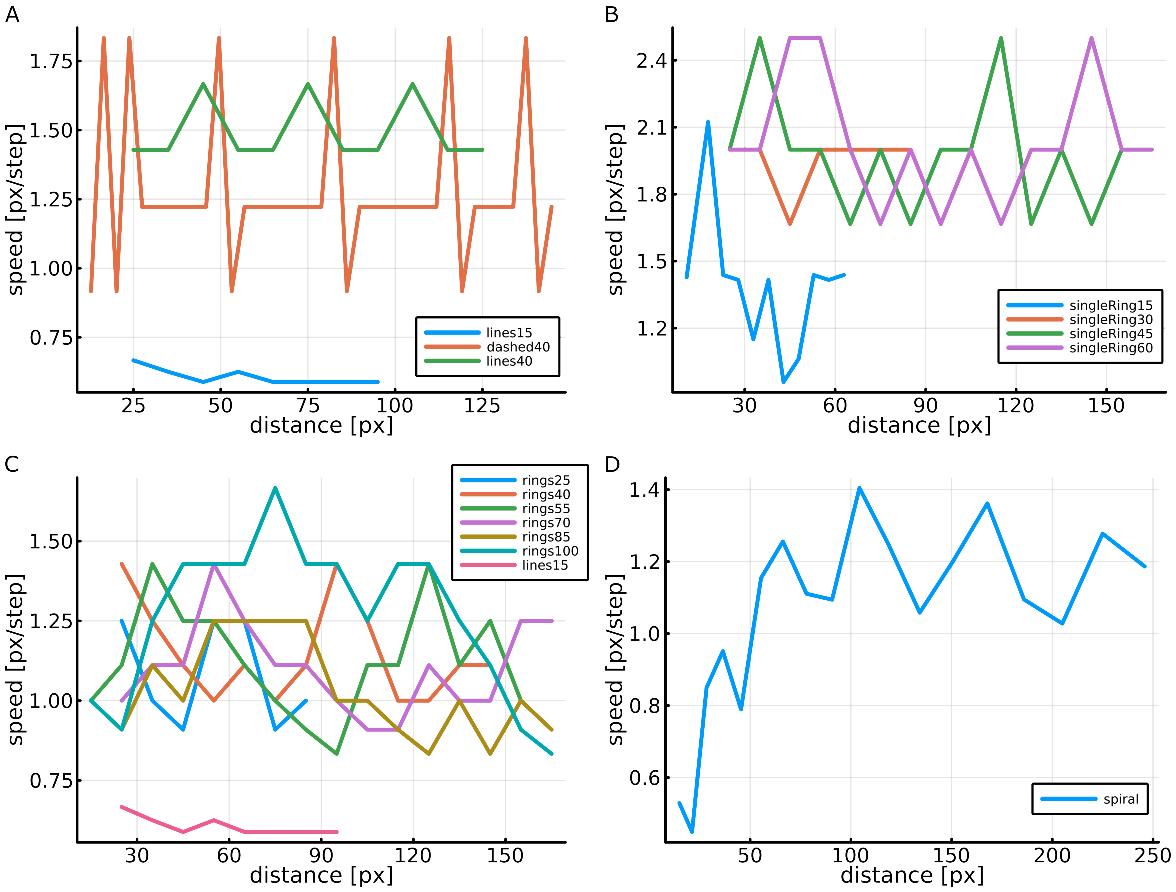


**Fig A. Incremental binding speed along more complex stimuli.**
Speed profiles for the different cases of stimuli measured for probe positions along the extent of the respective structure. Distance is measured as path length along the respective ring from the attentional seed point. (A) Speed profiles for `lines` and `dashed` stimuli. (B) Speed profiles for `singleRing` stimuli. (C) Speed profiles for `rings` stimuli. (D) Speed profile for `spiral` stimulus. Note, that `lines15` is the identical profile in both, A, C. Further note, that y-axes are scaled to the respective value ranges. This display extends the display in Fig 9E for how data samples for speed measurements are organized along contour distance.

**Table A:** Statistical values summarizing the tracing speeds for the additional complex stimuli experiments.

| **Stimulus** | **Number of probes** | **Median speed [px/step]** | **Mean speed [px/step]** | **Standard deviation [px/step]** |
|---|---|---|---|---|
| lines15 | 8 | 0.59 | 0.61 | 0.03 |
| rings25 | 7 | 1.00 | 1.08 | 0.16 |
| rings40 | 13 | 1.11 | 1.15 | 0.15 |
| rings55 | 16 | 1.11 | 1.11 | 0.17 |
| rings70 | 15 | 1.11 | 1.10 | 0.14 |
| rings85 | 15 | 1.00 | 1.03 | 0.15 |
| rings100 | 16 | 1.34 | 1.26 | 0.24 |
| singleRing15 | 11 | 1.42 | 1.39 | 0.30 |
| singleRing30 | 9 | 2.00 | 1.93 | 0.15 |
| singleRing45 | 15 | 2.00 | 1.98 | 0.26 |
| singleRing60 | 15 | 2.00 | 2.03 | 0.28 |
| spiral | 18 | 1.10 | 1.06 | 0.26 |
| dashed40 | 37 | 1.22 | 1.27 | 0.27 |
| lines40 | 11 | 1.43 | 1.49 | 0.11 |

spreading performed upon it (Sect 2.2.4). Additional experiments have been performed to see whether and how these findings generalize. This additional set of stimuli consists of lines that meet under different oblique angles and either intersect each other or not (Fig B). The set of stimuli relates to earlier experimental evidence in which (non-)human primates had to trace curves under conditions where lines would sometimes be meeting each other and either would intersect each other or not [1, 2]. Overall, the model generalizes to such configurations for a range of different angles under which the lines are meeting. Likewise, the model's limitations become visible for more extreme angles.

On the set of stimuli with intersections the angle was varied between 15 (almost parallel) and 165 degrees in steps of 15 degrees leading to almost a parallel and an "H"-shaped configuration, respectively. The crossing configuration was embedded into vertical line segments. In total, image sizes ranged from 229 $\times$ 229 pixels for 15 degrees to 179 $\times$ 179 pixels for 165 degrees intersection angle. While the length of the vertical line segments differed for the different intersection angles, the structure size (line width) and intensity values remained the same across the stimuli. To create these stimuli the same stimulus creation procedure as for the varied set of complex stimuli has been employed (cf. S1 Text). The line width was set to 1.0 pixel and the Gaussian standard deviation was set to $\sigma^p = 0.75$ pixels. The cutoff value has been set to 0.15, and the value range to $[0.0, 0.5]$.

Overall, the model generalizes to a considerable range of intersection angles. Out of the set of stimuli, the model was able to successfully perform incremental binding in the range of 60 to 135 degrees. More extreme intersection configurations led to fail cases, with two distinct error patterns. For large angles (150 and 165 degrees) the model stopped spreading right at the intersection node. In this case the orientation difference between the two intersecting lines is so small, that neurons residing in the respective orientation maps interfere strongly with each other as though they would

be parallel to each other (Fig 8). This effect takes place for a spatially extended region due to the large intersection angle. This prohibits further propagation of the binding signal. For small angles (15, 30, and 45 degrees) the model stopped at the point of branching out of the vertical line. There, the orientation difference between adjacent line elements is too large, such that no compatibility based on collinearity can be established and tracing stops. We suggest that the former issue can be solved by incorporating long-range base grouping filters into the model [3, 4, 5], while the latter one would require extending the base representation for curvatures [6] or end-stops and junctions [7, 8, 9].

On the set of non-intersecting stimuli each side of the stimulus configuration consists of vertical lines with a center component, which has slanted lines meeting under a certain opening angle (Fig B). The stimuli were created by mirroring one side of the configuration, but with a displacement of 8 pixels from each other, such that the lines do not touch each other. Opening angles have been varied between 45 and 135 degrees in steps of 15 degrees leading to stimulus sizes between $197 \times 183$ and $283 \times 269$ pixels, respectively, while keeping the structure size (line widths) constant between the stimuli.

The model performs incremental binding on a subset of the stimuli. If opening angles were large enough, i.e., the line continued without too abrupt changes in direction, tracing continued beyond the center non-crossing point (120 and 135 degrees). When the line continued beyond the center point under an acute angle, and therefore with a larger change in direction, tracing stopped and did not continue (angles of 105 degrees and smaller). This case resembles the one of intersecting lines from above for too large orientation differences between adjacent lines (small angles fail case). The same solution of extending the base representation for curvature [6] or end-stop and junction cells [7, 8, 9] applies.

## S2.3 Additional eccentricity experiments

The incremental binding model proposed in this work did not incorporate eccentricity-dependent receptive field size effects [10, 11]. Such eccentricity-dependent receptive field sizes would require a translation-variant filter application [12], which is different from a linear convolution operation employed by the model (cf. Sect 4.1). Here, we test the model for the influence of eccentricity-dependent effects in two reduced settings. These settings approximate eccentricity-dependent filtering to estimate effects on the model's computation regarding tracing speed and loss in spatial acuity.

Visual cortex receives retinal input relayed by the LGN. Primates possess a foveated retina with a high photoreceptor density close to the center (fovea) and a decreasing density with increasing eccentricity from the center (parafoveal and peripheral). This variation in photoreceptor density, together with cortical sampling leads to a non-uniform resolution of image space and altered perceptual properties in the periphery [13]. The resulting retinotopic visual representation in cortex can be described in a log-polar space [14, 15, 16].

As a result of the non-uniform resolution, visual acuity largely depends on the eccentricity of the visual location with respect to the foveal center [17]. While this eccentricity-dependent decline happens rather gradually, three regions of eccentricity are commonly distinguished, namely foveal ($0°$ to $1°$), parafoveal ($1°$ to $4 - 5°$), and peripheral ($\geq 4{-}5°$) regions. So far, stimuli for investigating phenomena of incremental

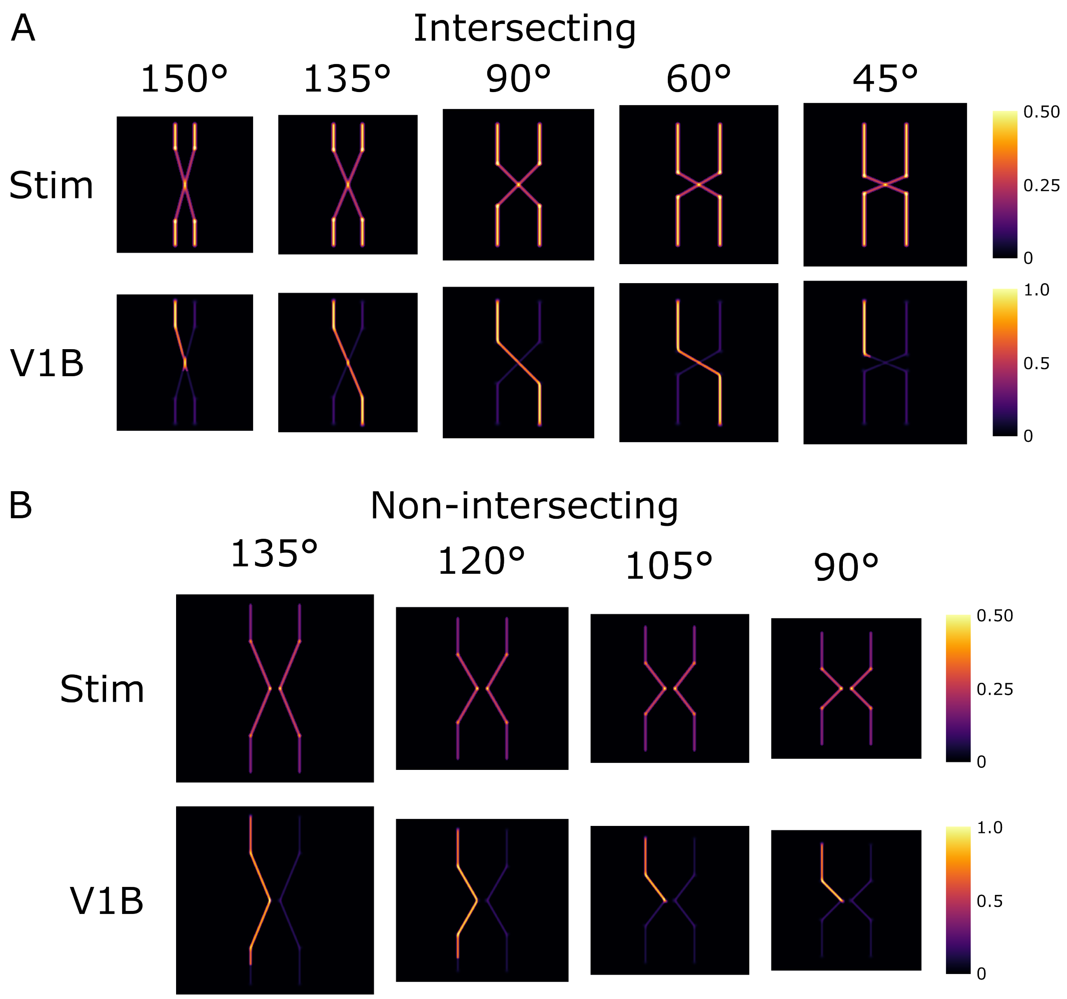


**Fig B. Line experiments for intersecting and non-intersecting curves under varying angles.**
Depicted are the stimuli (first row, third row) and the model states in terms of aggregated basal activity of V1 neurons after convergence (second row, fourth row) for a range of angles (columns). (A) Intersecting stimuli are depicted for angles from almost parallel line configurations (left, 150 degrees intersection angle) to almost "H"-like configurations (right, 45 degrees intersection angle). Image sizes are scaled to match the respective relative map sizes between experiments. (B) Non-intersecting stimuli for angles ranging from obtuse (left, 135 degrees opening angle) to more acute configurations (right, 90 degrees opening angle). Image sizes are scaled to match the respective relative map sizes between experiments.

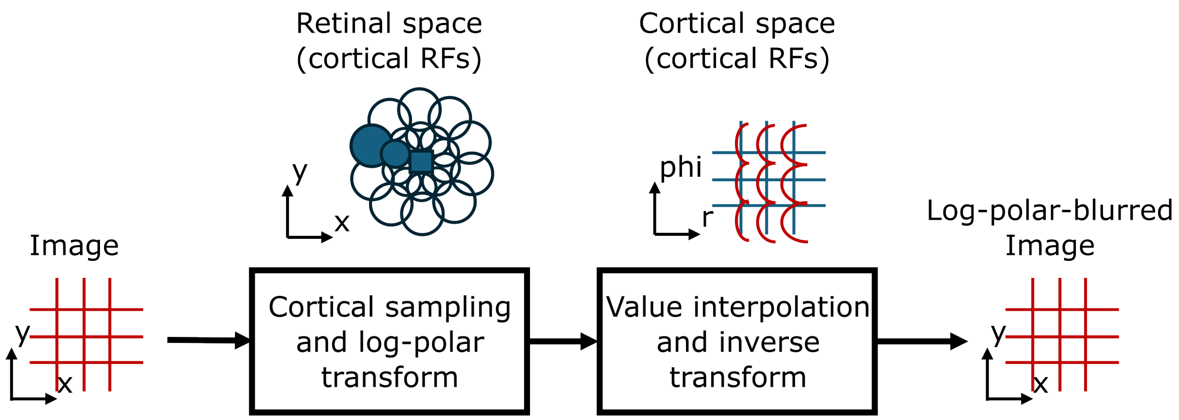


**Fig C. Log-polar transformation and back-projection to compute cortical responses in Cartesian space.**
Image transformation pipeline utilized to create log-polar-blurred images as input to the incremental binding model. An image in Cartesian coordinates (retinal space, red grid) is sampled by Gaussian kernels placed and scaled based on a cortical parametrization, such that the log-polar space ($r$, $phi$; cortical space, blue grid) is sampled equidistantly. Then, the cortical representation is evaluated at Cartesian image coordinates (red arcs) by value interpolation. This yields a log-polar-blurred image back-projected to equidistant pixels in Cartesian space.

grouping have largely been residing in (para-)foveal size ranges. Approximate maximal stimulus eccentricities were $5.9°$ [18], $4°$ [19], $4 - 8°$ [2] (estimated from stimuli shown in these displays, $2.8°$ mean RF eccentricity of measured neurons according to the text by the authors), and $5 - 9.5°$ [20]. Yet, some studies extended at least up to a certain point into the periphery, e.g., $9°$ and $13.5°$ [21] (eccentricities for their experiments 1 and 2, respectively), and $15°$ [22].

In general, it is not clear, how (incremental) binding proceeds in the periphery and models so far have not considered log-polar space representations. Furthermore, whether grouping processes operate in a similar fashion in the periphery as they do in (para-)foveal regions is an additional question on its own [23]. The recently proposed central-peripheral dichotomy, for example, assigns vastly different functional roles of central versus peripheral (visual) processing [24, 25]. Additionally, susceptibility to crowding from distractors would need further consideration [26].

We show to what extend the model results carry over to these eccentricities, how tracing time predictions may change for larger eccentricities and that additional mechanisms may be required for grouping under distortions arising from the far periphery. To this end, we employ a log-polar input transformation that mimics the eccentricity-dependent increase in receptive field sizes, the loss in spatial resolution and an increase in spatial blurring (Fig C, see Sect S2.4 for details).

### S2.3.1 Eccentricity-dependent characteristics of tracing speed

Additional simulations have been performed to understand how the presented incremental binding mechanism will be affected by eccentricity-dependent increases in receptive field sizes (Fig D). The model was tested on a simple stimulus consisting of a target line and a distractor extending towards the eccentricity similar to studies in monkeys [19]. As for the main experiments (Fig 6), lesion studies have been performed

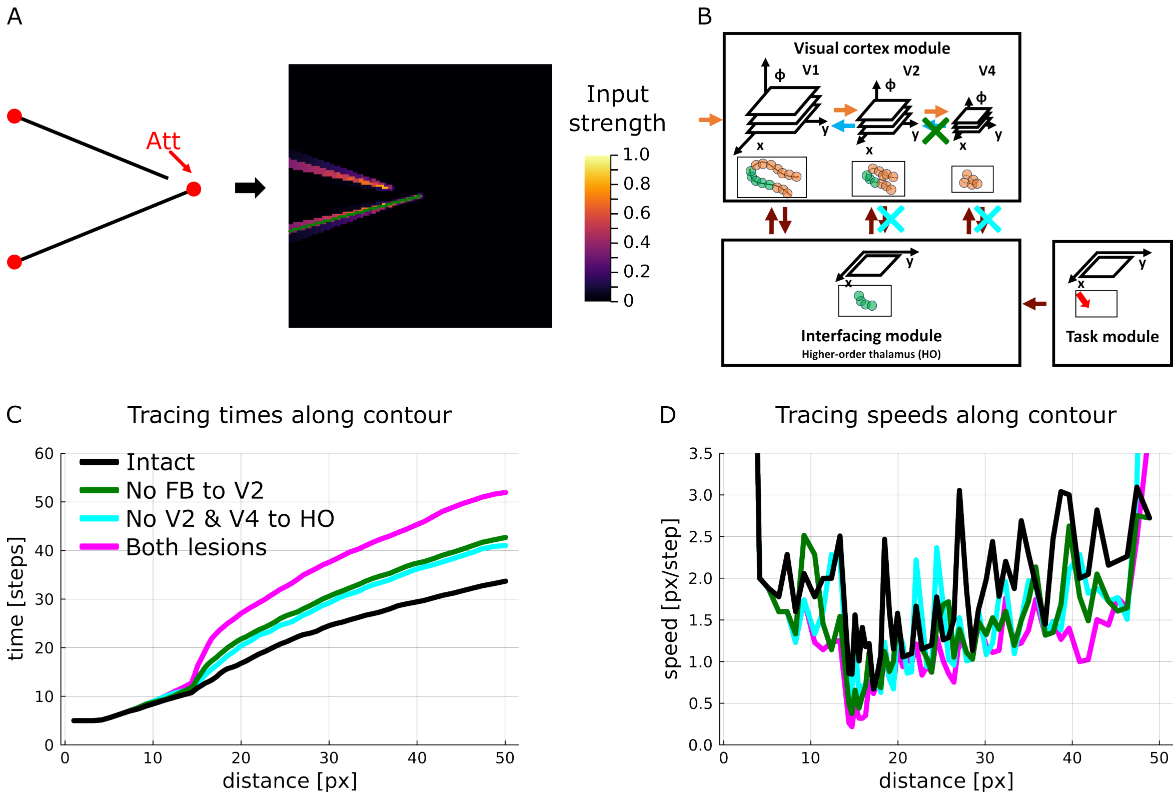


**Fig D. Experiments on eccentricity-dependent receptive field size effects and lesions.**
(A) Stimulus configuration used for investigating eccentricity-dependent receptive field size effects (left). Actual model input after application of log-polar transformation (right; see Sect S2.4 for details). The green line overlaid onto the stimulus indicates the readout positions of the neural signals. (B) Based on the modular architecture specific connectivity patterns can be lesioned to investigate their functional role (cf. Fig. 6 for further lesion experiments). (C) Tracing time grows sub-linearly with eccentricity and increases if neurons with larger receptive fields are lesioned. Removing either feedback from model area V4 to V2 or projections to the interfacing module (higher-order thalamus, HO) results in a similar increase of tracing time. Applying both lesion cases at the same time results in a further increase of tracing time. (D) Tracing speed increases with increasing eccentricity. For a parafoveal region tracing speed is rather constant. At the point closest to the distractor line, a reduction of tracing speed is visible for all investigated cases. For larger eccentricities a trend of linearly increasing tracing speed is noticable. While the individual and combined lesion cases result in rather similar tracing speed profiles, the intact model reaches higher speeds faster. This shows that the model requires both pathways (cortico-cortical feedback and cortico-thalamic projections) from higher levels of the visual cortex module to efficiently leverage the larger receptive field sizes of these levels.

on this stimulus configuration to investigate the contribution of higher-level representations of the visual cortex module to the tracing times and to the tracing speed profile (Fig D).

For a more complex stimulus on a Cartesian grid, i.e., without eccentricty-dependent effects, a section-wise linear increase in tracing times, and, thus, section-wise constant tracing speeds have been observed (Fig 6A and 6C). In contrast, the eccentricity-scaling on the more simplistic stimulus here exhibits sub-linear increases in tracing speeds for larger eccentricities, and, thus, non-constant and growing tracing speeds (Fig D). As a result, larger distances along the stimulus are covered by attentional up-modulation during the same amount of time for stimulus sections further towards the periphery.

Notably, the overall tracing time and speed is strongly dependent on a joint effect of projections from neurons with large receptive field sizes at large eccentricities. If feedback projections from model area V4 to V2 and from both areas to the higher-order thalamic (HO) module are lesioned, tracing time drastically increases and tracing speed is reduced. While recovering each of the pathways results in an improvement of tracing performance (no feedback from V4 to V2, or no projections from V2 and V4 to HO), only both pathways combined recover the fast tracing of the intact model. These simulations demonstrate how tracing times, and, thus, reaction times may grow sub-linearly with increases in eccentricities for simple stimuli.

### S2.3.2 Eccentricity-dependent loss of resolution

The model was tested on log-polar-blurred versions of the stimuli with varying target-distractor distance described in Sect 2.2.2 (Fig 4). As a comparison case the model was tested as well on the identical stimuli without application of this retino-cortical transformation. Stimuli for these simulations were created in two steps. At first the Cartesian images have been created using the implementation from the respective varying target-distractor distance panels (cf. S1 Text). Afterwards, the log-polar transformation and back-projection has been applied to obtain the log-polar-blurred image versions (Fig C).

The different input transformations were tested for a stimulus version with a short fraction of narrow distance and a larger fraction (25% and 50% of the overall stimulus size, respectively; Fig E). For the stimulus with the short fraction all input transformations yield a similar result of tagging the target contour with attention. For the larger fraction, the results differ per model. The untransformed image result in successful tracing, as expected from the main paper's results (Fig 5). The retino-cortically transformed images result in either tracing that starts on the target but continues on the distractor (nearest neighbor version) or starts on the target and stops at a certain eccentricity when blur becomes too severe (linear interpolation version). Different from the other tested stimuli, for this stimulus an input-transformation-dependent effect becomes visible. The interaction seems to depend on line distance and eccentricity. There, the difference in outcomes depends on the narrow fraction of the line where target and distractor are close to one another, but the impact only takes effect after the lines are blurred enough by a large radial distance from the center of fixation.

Taking these results together, it is likely that tracing stimuli further toward the periphery can proceed faster (Sect S2.3.1), but also becomes more prone to incorrect grouping of stimulus elements.

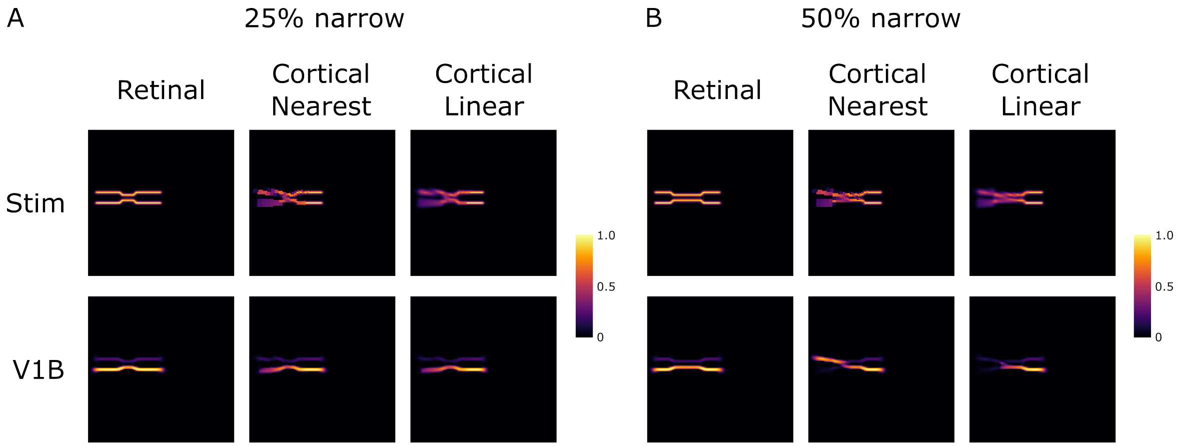


**Fig E. Simulation results on stimuli with varying target-distractor distance under different input transformations.**
Stimulus depiction (first and third column) for no input transformation (Retinal, first row), and retino-cortical transformation and back-projection with nearest neighbor interpolation (Cortical Nearest, second row) and linear interpolation of retinal image pixel values (Cortical Linear, third row). Simulation outcomes in terms of basal V1 activity aggregated over orientation channels (second and fourth column). (A) The simulation for a narrow fraction of 25% yields a successful tracing of the target contour irrespective of the input transformation. For retino-cortically transformed variants blurring becomes more severe with eccentricity and yields a less pronounced up-modulation for the line end. (B) The simulation for a narrow fraction of 50% yields a successful tracing of the target contour only for the untransformed (Retinal) case. For the nearest-neighbor interpolated retino-cortically transformed image (Cortical Nearest) an originally successful tracing continues onto the distractor curve beyond a certain eccentricity of the narrow distance segment of the stimulus. The linear interpolation version of the retino-cortical transformation (Cortical Linear) tracing of the target contour stops on the narrow distance segment at a certain eccentricity where blurring becomes too severe.

## S2.4 Methods for additional eccentricity experiments

Here, we focus on the impact of uneven distribution of cortical resolution with respect to retinal input. To answer this question, we employ a log-polar transform that captures the main hallmarks of the retino-cortical spatial input transformation (see [15, 27] for further details on the log-polar transform method). Subsequent back-projection of the resulting cortical values retrieves a Cartesian space again compatible with the proposed model (Fig C).

First, a cortical population is parametrized in log-polar space and a fixation cue location is determined. The log-polar space is sampled equidistantly in angle and log-radius leading and receptive field sizes are approximated by Gaussian kernels which scale in size with log-radius. This way, an eccentricity dependent resolution of images will be established. To keep the transformation under application of the $log$-function sound, radii below a certain threshold are kept in Cartesian representation and cortical neurons sample from these position in a 1-to-1 correspondence. As a result, no sub-sampling or blurring happens at the foveal center, but increasingly takes effect for larger distances.

Then, a Cartesian input image is sampled by this cortical population, where the input's values are evaluated at the cortical neuron positions. This evaluation is performed by interpolation using the Gaussian receptive fields obtained above.

Next, the cortical values are back-projected to the original retinal input positions in Cartesian space re-evaluating them at the original pixel positions. To perform this re-evaluation, we consider two options: nearest-neighbor sampling, where the closest cortical neuron activity is mapped to the pixel location, and linear interpolation, where the activities of the cortical neurons surrounding the pixel location are linearly interpolated.

This cortical projection and back-projection recovers an image of equidistant pixel locations usable with the proposed image-computable model architecture. Yet, under this eccentricity-dependent cortical sub-sampling and back-projection the information loss, i.e., blurring, is similar to the one that a log-polar-operating architecture would need to accommodate for (Sect S2.3.2). As a consequence, this procedure provides a first estimate of the effect of a loss of resolution under peripheral stimulus presentation on incremental binding outcomes in the model.

The size of the cortical population for parametrizing the transformation was kept constant at $127 \times 127$ neurons.

Representing these interpolated values in the intermediate cortical log-polar space (cf. Fig C) would lend itself to further processing in a model cortical hierarchy with log-polar modeled filter kernels to accommodate for the distorted space and a respective space-variant filtering approach with an eccentricity-dependent increase in receptive field sizes [28, 29, 30]. This investigation is left for future work. Yet, for simplistic cases an approximate solution for the impact of such eccentricity-dependent receptive field size scaling can be recovered with the present approach (Sect S2.3.1). To this end, we employ the above described algorithm with a slight adaptation. Instead of back-projecting onto the original retinal input positions that formed a Cartesian grid, the back-projection happens onto a rectangular grid of values which maintains the log-scaling from the cortical representation. In order to achieve this, the retinal coordinates are represented in a polar coordinate system at first. There, the radial component of each coordinate is rescaled to linearly sample from the range of eccentricities provided

by the cortical log-polar representation. Afterwards, there log-scaled coordinates are then used for the back-projection of the cortical log-polar representation. The effect of this non-linear increase in radial distances of input positions propagates via the feed-forward projections of the visual cortex module onto the receptive fields of each level. This way, even with uniformly sampling their lower-level inputs, receptive fields of higher levels of the visual cortex module will be distorted and increase with increasing eccentricity [31]. To accommodate for the resulting more dispersed projections, the feedback strengths $\lambda^V$ have been increased for this simulation from 15.0 to 30.0 (cf. Eq 12 and Table A in S1 Text).

# References


[1] Houtkamp R, Spekreijse H, Roelfsema PR. A gradual spread of attention during mental curve tracing. Perception & Psychophysics. 2003;65(7):1136–1144. doi:10.3758/BF03194840.

[2] Pooresmaeili A, Roelfsema PR. A Growth-Cone Model for the Spread of Object-Based Attention during Contour Grouping. Current Biology. 2014;24(24):2869–2877. doi:10.1016/j.cub.2014.10.007.

[3] Grossberg S, Mingolla E. Neural dynamics of perceptual grouping: Textures, boundaries, and emergent segmentations. Perception & Psychophysics. 1985;38(2):141–171. doi:10.3758/BF03198851.

[4] Li Z. A Neural Model of Contour Integration in the Primary Visual Cortex. Neural Computation. 1998;10(4):903–940. doi:10.1162/089976698300017557.

[5] Hansen T, Neumann H. A recurrent model of contour integration in primary visual cortex. Journal of Vision. 2008;8(8):8–8. doi:10.1167/8.8.8.

[6] Rodríguez-Sánchez AJ, Tsotsos JK. The Roles of Endstopped and Curvature Tuned Computations in a Hierarchical Representation of 2D Shape. PLOS ONE. 2012;7(8):e42058. doi:10.1371/journal.pone.0042058.

[7] Weidenbacher U, Neumann H. Extraction of Surface-Related Features in a Recurrent Model of V1-V2 Interactions. PLOS ONE. 2009;4(6):e5909. doi:10.1371/journal.pone.0005909.

[8] Marić M, Domijan D. Neural dynamics of spreading attentional labels in mental contour tracing. Neural Networks. 2019;119:113–138. doi:10.1016/j.neunet.2019.07.016.

[9] Domijan D, Marić M. A multi-scale neurodynamic implementation of incremental grouping. Vision Research. 2022;197:108057. doi:10.1016/j.visres.2022.108057.

[10] Gattass R, Gross CG, Sandell JH. Visual topography of V2 in the macaque. Journal of Comparative Neurology. 1981;201(4):519–539. doi:10.1002/cne.902010405.

[11] Gattass R, Sousa A, Gross C. Visuotopic organization and extent of V3 and V4 of the macaque. The Journal of Neuroscience. 1988;8(6):1831–1845. doi:10.1523/jneurosci.08-06-01831.1988.

[12] Ahrns I, Neumann H. Space-Variant Image Processing. In: Baratoff G, Neumann H, editors. Dynamische Perzeption. vol. 9 of Proceedings in Artificial Intelligence. Köln: Infix Verlag; 2000. p. 203–206.

[13] Strasburger H, Rentschler I, Jüttner M. Peripheral vision and pattern recognition: A review. Journal of Vision. 2011;11(5):13. doi:10.1167/11.5.13.

[14] Schwartz EL. Spatial mapping in the primate sensory projection: Analytic structure and relevance to perception. Biological Cybernetics. 1977;25(4):181–194. doi:10.1007/BF01885636.

[15] Ahrns I, Neumann H. Space-variant dynamic neural fields for visual attention. In: Proceedings. 1999 IEEE Computer Society Conference on Computer Vision and Pattern Recognition (Cat. No PR00149). Fort Collins, CO, USA: IEEE Comput. Soc; 1999. p. 313–318.

[16] Fazl A, Grossberg S, Mingolla E. View-invariant object category learning, recognition, and search: How spatial and object attention are coordinated using surface-based attentional shrouds. Cognitive Psychology. 2009;58(1):1–48. doi:10.1016/j.cogpsych.2008.05.001.

[17] Loschky L, McConkie G, Yang J, Miller M. The limits of visual resolution in natural scene viewing. Visual Cognition. 2005;12(6):1057–1092. doi:10.1080/13506280444000652

[18] Jolicoeur P, Ullman S, Mackay M. Curve tracing: A possible basic operation in the perception of spatial relations. Memory & Cognition. 1986;14(2):129–140. doi:10.3758/BF03198373.

[19] Roelfsema PR, Lamme VAF, Spekreijse H. Object-based attention in the primary visual cortex of the macaque monkey. Nature. 1998;395(6700):376–381. doi:10.1038/26475.

[20] Roelfsema PR, Khayat PS, Spekreijse H. Subtask sequencing in the primary visual cortex. Proceedings of the National Academy of Sciences. 2003;100(9):5467–5472. doi:10.1073/pnas.0431051100.

[21] Jeurissen D, Self MW, Roelfsema PR. Serial grouping of 2D-image regions with object-based attention in humans. eLife. 2016;5:e14320. doi:10.7554/eLife.14320.

[22] Khayat PS, Pooresmaeili A, Roelfsema PR. Time Course of Attentional Modulation in the Frontal Eye Field During Curve Tracing. Journal of Neurophysiology. 2009;101(4):1813–1822. doi:10.1152/jn.91050.2008.

[23] Hess R, Field D. Integration of contours: new insights. Trends in Cognitive Sciences. 1999;3(12):480–486. doi:10.1016/S1364-6613(99)01410-2.

[24] Zhaoping L. A new framework for understanding vision from the perspective of the primary visual cortex. Current Opinion in Neurobiology. 2019;58:1–10. doi:10.1016/j.conb.2019.06.001.

[25] Zhaoping L. Peripheral and central sensation: multisensory orienting and recognition across species. Trends in Cognitive Sciences. 2023;27(6):539–552. doi:10.1016/j.tics.2023.03.001.

[26] Rosenholtz R. Capabilities and Limitations of Peripheral Vision. Annual Review of Vision Science. 2016;2(1):437–457. doi:10.1146/annurev-vision-082114-035733.

[27] Baratoff G, Schönfelder R, Ahrns I, Neumann H. Orientation Contrast Detection in Space-Variant Images. In: Goos G, Hartmanis J, Van Leeuwen J, Lee SW, Bülthoff HH, Poggio T, editors. Biologically Motivated Computer Vision. BMVC 2000. Lecture Notes in Computer Science. vol. 1811. Berlin, Heidelberg: Springer Berlin Heidelberg; 2000. p. 554–563.

[28] Mallot HA, Seelen WV, Giannakopoulos F. Neural mapping and space-variant image processing. Neural Networks. 1990;3(3):245–263. doi:10.1016/0893-6080(90)90069-W.

[29] Wallace RS, Ong PW, Bederson BB, Schwartz EL. Space variant image processing. International Journal of Computer Vision. 1994;13(1):71–90. doi:10.1007/BF01420796.

[30] Fischl B, Cohen MA, Schwartz EL. The Local Structure of Space-variant Images. Neural Networks. 1997;10(5):815–831. doi:10.1016/S0893-6080(96)00125-6.

[31] Harvey BM, Dumoulin SO. The Relationship between Cortical Magnification Factor and Population Receptive Field Size in Human Visual Cortex: Constancies in Cortical Architecture. The Journal of Neuroscience. 2011;31(38):13604–13612. doi:10.1523/jneurosci.2572-11.2011.
