# Supplementary material for: A model of thalamo-cortical interaction for incremental binding in mental contour-tracing
Source: PLoS Comput Biol. 2025 May 8;21(5):e1012835. doi: 10.1371/journal.pcbi.1012835 (PMC12061125; doi:10.1371/journal.pcbi.1012835)
Supplement: Attachment — Submitted filename: ResponseToReviewers.pdf [file pcbi.1012835.s003.pdf]

General response to reviewers

Dear Reviewers,

thank you very much for your detailed and highly thoughtful comments on the initial version of our manuscript. We value your contribution that helped us with increasing the relevance of our work, as well as with improving the clarity of its presentation.

Among all the changes made to the manuscript there are a few to be highlighted:

- We incorporated your feedback about the unclear relationship between our model and evidence from biology and provide a detailed discussion, especially of the relevance and putative role of higher-order thalamus as an interfacing module in performing incremental binding.
- To better understand if the model can generalize to stimuli which are more complex than straight lines, such as curved lines, dashed lines, or different kinds of intersecting lines, we conducted extensive additional experiments and provide additional discussion thereof. Remarkably, the model was able to generalize to a great proportion of the tested stimuli in the different experiments – without changing any model parameters.
- We greatly improved the presentation of our work in terms of textual descriptions and illustrations. To make our work more accessible we improved the conceptual depictions and added further diagrams to highlight how the performed simulation and complexity considerations integrate with each other and how the model is working internally. An important highlight in this regard constitutes the depiction of relative receptive field and kernel sizes for the different feedforward and feedback connections (Fig. 12). We hope this figure further clarifies how the model connects to biological evidence and helps to avoid misunderstandings regarding the employed scale space approach by convolution and resizing in our model.

Below we provide a detailed list of answers to all your comments, per reviewer. We further structured our responses by providing references to the respective pages of the revised version of our manuscript (**[black]**) and of the version of our manuscript that highlights the changes since our initial submission (**[red]**). Supporting information material is denoted by an additional “Sx:” in front of the page number referring to the respective supplement (Sx).

Thank you for your consideration.

Kind regards,
Daniel Schmid & Heiko Neumann

Response to reviewer 1

We thank you for your suggestion to provide more comprehensive testing of our model to better understand the model's capabilities and limitations, and to better explain our approach and its relation to biology. We value your detailed suggestions for additional test configurations, which we incorporated throughout the manuscript.

Regarding 1. Stimulus configurations

- *Model behavior on curves meeting and (not) intersecting under different angles (e.g., like this ><):*
  To answer this question we extended our implementation for another set of (non-)intersecting curves meeting under different angles and tested the model on a range of respective parametrizations. The results are presented as additional material in the Appendix S2.2 **[S2:1-5]**. The model generalizes to a range of angles for intersecting and non-intersecting lines (without changing any model parameters). Likewise, limitations for more extreme angles become apparent and we highlight the necessity for extending the model, e.g., by incorporating neurons selective to end-stop and/or junction configurations Sect. 3.5.1 **[38][40]**. These simulations provide first insights to what extend the model generalizes to stimuli similar to the configurations from Houtkamp et al. (2003, Perception & Psychophysics) mentioned in your comments.

- *Model behavior on curves of various degrees of curvatures:*
  We extended our implementation for another set of curves forming rings around a common center point, similar to experiments conducted with human observers (Jolicoeur et al., 1991, JEP: HPP). These results are presented as additional experiments in Sect. 2.2.5 **[18-20][19-21]** with further material being provided in the Appendix S2.1 **[S2:1-3]**. In general, the model can trace these contours as individual rings and under the presence of close-by distracting rings. The effect reported in the literature for human observers, that with increasing curvature an increase in tracing time is required, is explained to some extend by the model. For high curvatures such effect is visible. Yet, for smaller curvatures no further decline in tracing speed is detectable. We discuss this as a limitation of the model's base representation and the possible extension towards incorporating curvature-selective neurons into the model Sect. 3.5.1 **[38][40]**.

- *Model behavior on broken curves:*
  We now provide a set of simulations on tracing dashed contours with small gaps between collinear line elements as part of the additionally conducted experiments. The results from these experiments are presented in Sect. 2.2.5 **[18-20][19-21]** and further details in the Appendix S2.1 **[S2:1-3]**. We find a similar overall tracing speed for dashed line configurations compared to solid lines with identical target-distractor distances. These findings are compatible with the findings from Houtkamp & Roelfsema (2010, JEP: HPP) mentioned in your comments. Additionally, we highlight the model's limitations regarding broken lines with larger gaps and suggest an extension of the base representation for long-range grouping filters to cope with such more demanding stimuli Sect. 3.5.1 **[38][40]**.

Regarding 2. Biological considerations

- *Detailed description of relation to thalamic oscillations:*
  We extended our discussion section for a more detailed consideration of the nature of thalamo-cortical interactions and the role and properties of higher-order thalamic (pulvinar) function Sects. 3.1.4-3.1.7 **[30-33][32-35]**. We provide an overview of the role of cortical oscillations during attentional processing and relate the presented model to theories about rhythmic attentional processing (Fiebelkorn & Kastner, 2019, Trends in Cognitive Sciences). In short, we outline that the model thalamic neurons' gating role for cortico-cortical context integration could serve as a mechanism to alter functional connectivity. Therefore, dependent on the attentional state of the system, i.e., performing incremental binding or another mental operation, theta-band oscillations within the functional fronto-parietal network would be modulated and dampened during prolonged attentional processing, similar to the reported evidence (Fiebelkorn et al., 2019, Nature Communications). Since to date no measurements exist specifically testing for the impact of incremental grouping on brain-wide oscillations, our considerations likewise form model predictions Sects. 3.1.5 & 3.4 **[31-32;36-37][33-34;39]**.

- *Relation to attentional and non-attentional units (A-/N-units) as measured in monkey V1 (Pooresmaeili et al., 2010, Journal of Neuroscience):*
  We now properly acknowledge the existence of A- and N-units and discuss the existing evidence in this regard Sect. 3.1.3 **[30][31-32]**. We draw relations to the model and note to the reader that it is based on A-unit processing. Additionally, we note that the employed neuron model description is in principle generic enough to be extensible towards N-unit behavior.

- *Spatial size of feedforward and feedback projections and relation to neuroanatomy (Angelucci et al., 2002, Journal of Neuroscience):*
  We refined and extended the description of our modeling approach to clearly communicate to the reader which size relations apply between the modeled connection types **[40-42][43-45]**. This relationship is now visualized in terms of Fig. 12 **[41][44]**. The presented feedforward, lateral inhibitory and feedback connections follow the typical increase in projection sizes as known from biology (Angelucci et al., 2002, Journal of Neuroscience). These respective size proportions were already present in the original model description as per our initial submission, i.e., no model parameters were changed to achieve this effect. We acknowledge for the unclear specification and apologize for this potential confusion. We now make clear to the reader that these relations arise from the combination of applying convolution kernels of equal size, but on different levels of a scale space which is formed by resizing neural feature maps after application of the convolution operation.

Regarding 3. Model description

- *Explicit report of the derivation of the steady-state approximation:*
  A section has been added as Appendix S1.1 clearly describing how the steady-state approximation is derived for the employed model equations **[S1:1-2]**. We provide the necessary detail to the reader for better understanding the approach and clearly state the

exact equations that have been used for simulating the model for the different experiments Sect. 4.2.1 **[45][49-50]**.

- *Observations about criticality of model parameters:*
  We extended the description of our simulation details and now provide a detailed description of observations made regarding the criticality of individual model parameters for proper model function S1.2 **[S1:2-4]**.

- *Missing dot above temporal derivatives of state variables:*
  Mathematical equations have been closely screened for notational issues and all temporal derivatives now are properly regarded by a dot above the respective state variable Sect. 4.1.2 & 4.1.3 **[43-45][46-48]**. We apologize for this oversight in our initial submission.

Response to reviewer 2

We thank you for clear and thorough feedback for how to improve the presented work to more closely establish a link with biological evidence and to become more relevant for a broader range of more realistic visual scenes. We are also thankful for the list of additional comments that greatly helped us to improve the manuscript further, such as incorporating a figure that relates specific contributions of the work to Marr's levels.

Regarding 1. Scaling response field size with eccentricity

- *Concern about the model (not) accounting for eccentricity-dependent receptive field size scaling and that the model could only be working for stimuli of a limited, small size:*
  We now acknowledge the fact that the model is operating on a Cartesian coordinate system with equidistant pixels and constant input resolution (Sect. 3.1.1) **[29][30-31]**. Additionally, we note that many of the reported earlier neurophysiological and behavioral experiments were conducted for (para-)foveal eccentricities and argue that for these eccentricities the Cartesian representation provides a viable approximation. To further test the impact of degradation of visual acuity with increased eccentricity we conducted an additional experiment and report the outcomes in the Appendix S2.3 **[S2:4-8]**. There, it becomes visible that only for larger eccentricities model behavior becomes affected by the decrease in spatial resolution. We further mention these limitations in the Discussion (Sect. 3.1.1) **[29][30-31]** and likewise note that the relationship between incremental grouping and peripheral vision isn't as clear yet as for (para-)foveal viewing ranges (S2.3) **[S2:6]**.

- *Relevance with respect to more realistic visual stimuli:*
  We provide a range of additional model simulations on more realistic visual stimuli. These simulation results can be found in Sect. 2.2.5 (Fig. 9) **[18-20][19-21]** and Appendix S2 (Fig. S2) **[S2:2]**. From these simulations we deduce the relevance of the modeling results for a broad class of stimuli and that the model is generalizable towards more realistic visual stimuli, such as the Pathfinder14 example (Fig. 9, f, g) **[19][20]**, which combines features of broken lines, locally changing curvatures, and cluttered scenes.

- *Link between visual areas and model scales:*
  A newly added figure now illustrates how scales and areas in the model relate to each other (Fig. 12) **[41][44]**. There, it becomes visible that the model's spatial scale increases along the hierarchy of model visual areas from V1 to V4 (Fig. 12, c).

Regarding 2. Unclear role of the interface module

- *Need for interfacing module:*
  The conducted complexity consideration highlights different potential connectivity patterns where each of them could provide a functioning *implementation* of the incremental binding *algorithm* under the growth-cone hypothesis (Fig. 10, b) **[22][23]**. From this point of view, no immediate need for an interfacing module arises. Yet, by means of the complexity considerations we motivate why an interfacing module provides the most efficient

implementation among the alternatives regarding the number of connections required to implement the incremental binding algorithm and being able to efficiently incorporate attentional control signals (Fig. 11; Sect. 2.3.5) **[26;27-28][27;29]**. The manuscript now further highlights this argument that the interfacing module together with the proposed interaction scheme provides a *normative* account. Therefore, a figure now prominently illustrates the interrelation between these algorithmic and computational arguments on the one side and the implementation of the algorithm on the other (Fig. 2) **[5][5]**.

- *Impact of lesioning projections from V2 and V4 to the interfacing module on the growth-cone-like tracing speed variations:*
  The manuscript now better motivates the role of the lesioning experiments within the investigation (Sect. 2.2.3) **[12-15][13-16]**. Specifically, lesioning of the cortico-cortical feedback connections from V4 to V2 show that growth-cone-like properties would (almost) vanish, although the interaction of these areas with the interfacing module would still be kept intact (Fig. 6, c) **[13][14]**. V2 itself wouldn't receive any up-modulating feedback from V4 anymore due to the lesioning. Yet, V2 and V4 still can provide input to the interfacing module on a larger scale. This would in principle allow gates to open more quickly for context integration in V1 and provides the question of why, then, the tracing speed profile flattens, and growth-cone behavior is abolished. Besides open gating signals, the other important component for up-modulation of a model neuron is the availability of strong enough contextual evidence. In case of V1 this evidence is provided from V2, which remains at lower, baseline activity due to the lesioning. As a result, contextual evidence accumulation takes longer than for the intact model and tracing speed reduces to almost pixel-by-pixel tracing behavior (Fig. 6, c).

- *Need for separating out task and interfacing modules instead of the interfacing module being a self-contained source of attentional seeds:*
  The paper now clearly states that no such explicit need exists from a *computational* point of view, but that this provides a reasonable modeling assumption given evidence about the role of different cortical areas in attentional control (Sect. 3.1.8) **[33-34][35-36]**. Such attentional control is synonymous with providing the initial seed of attention to the proposed incremental binding mechanism, as otherwise the incremental binding process wouldn't be initiated.

Regarding 3. Loose link between model and biology

- *Relation between the interfacing module and higher-order thalamus:*
  The manuscript now better motivates the proposal of why higher-order thalamus could serve the role of an interfacing module during incremental binding. To this end, a newly added figure relates different contributions of the work to Marr's levels to better communicate how the complexity considerations integrate with the model implementation (Fig. 2) **[5][5]**. This integrated argument of simulation results and normative account puts forth the idea of an interfacing module. We now mention additional literature and discuss the causal role of higher-order thalamic regions in attentional processing to further motivate our prediction, that higher-order thalamus could fit the role of such interfacing module processing (Sects. 3.1.4 & 3.1.5) **[30-32][32-34]**.

- *Apical and basal integration site distinction:*
  The initial places in the manuscript where the concept of apical and basal integration sites is mentioned now contains a reference (Larkum, TINS, 2013). These places are the caption of Fig. 3 **[8][8]** and the model description (Sect. 2.1) **[9][9]**.

- *The paper's contribution to evidence of thalamo-cortical interactions in incremental binding:*
  The paper doesn't contribute experimental evidence but provides a modeling study together with further theoretical considerations. To better account for this fact, we adjusted the title of the manuscript to include "A model of…". We hope this prevents unwanted misunderstandings for potential readers of the manuscript in the future. Further, we improved the manuscript along all the suggested resolution options from your comments:
  - *Better support the link between the model and biology:*
    The paper now contains extensive references to experimental literature suggesting a causal role of higher-order thalamus in attentional processing (Sects. 3.1.4 & 3.1.5) **[30-32][32-34]**. Additionally, the manuscript acknowledges the possibility of alternatives for the biological substrate of the interfacing module and discusses an example (Sect. 3.1.8) **[34][36]**.
  - *Mentioning that parallels *may* exist during the discussion and that we make testable predictions that remain to be tested:*
    The discussion now explicitly mentions that our proposal about the role of higher-order thalamus poses a *model prediction* (Sects 3.1, 3.1.4) **[29;30][30;32]**. Furthermore, the manuscript now contains a dedicated list of specific model predictions (Sect. 3.4) **[36-37][39-40]**.

Regarding further comments on the manuscript

- *Unclear meaning of "complicated" objects in author summary:*
  We improved the usage of terminology and enhanced clarity throughout the manuscript. The mentioned cases of "complicated" objects was replaced at the respective place the author summary **[2][2]** by a more meaningful description. Likewise, the terminology had been adjusted further in the Introduction (Sect. 1) **[3-7][3-7]**. We apologize for the inexact writing.

- *Generalization to other stimuli and discussion thereof:*
  Model simulations have been extended based on suggestions from you and other reviewers. These extended results provide further insights into how far the model generalizes to other stimuli, e.g., consisting of broken line elements (Fig. 9, a), curvatures (Fig. 9 b, c), curves changing orientation continuously (Fig. 9 d), or combinations thereof Fig. 9 f, g). These insights are then further reviewed in the Results (Sect. 2.2.5) **[18-20][19-21]** and elaborated upon in the Discussion (Sects. 3.5.1 & 3.5.2) **[37-38][40-41]**.

- *More accurate description of previous work and clarification of generality of the findings:*
  The Introduction (Sect. 1) now contains a more extensive and accurate description of previous work that shows how general the findings about incremental grouping are, that they cover contour-, as well as region-based paradigms, and that they were obtained from (non-)human primate subjects Sect. 1 **[6][6]**.

- *Mentioning of the species from which referenced experimental findings were obtained (Fig. 1, introduction, and discussion):*
  The species from which referenced experimental findings were obtained are now clearly stated at the respective places of the Introduction (Sect. 1) **[6][6]**, Fig. 1, a **[4][4]**, and mention at the beginning of the Discussion, that we model *primate* brain (Sect. 3.1) **[29][30]**.

- *Scale of operation of the interfacing module:*
  We introduced an additional figure that illustrates relative projection sizes in relation to the scale of operation of the interfacing module (Fig. 12, b) **[41][44]**. Additionally, we now mention relative scales of operation for the different representations (V1, V2, V4, and HO) in the Methods (Sect. 4.1.1) **[40-42][43-45]**.

- *Potential confusion of curves lying within a common receptive field of the interfacing module*:
  While, in principle, such a confusion of curves lying within a common receptive field in the interfacing module would be possible, it never was encountered during the main experiments. Additionally conducted experiments with stimuli exhibiting more complex features, such as curvatures or broken lines, further provide evidence that this model behavior is rather the rule than the exception. Only if contours cannot be resolved as individual elements anymore, e.g., due to extensive blur, such target-distractor confusion and a spill-over of the attentional label may be encountered. This is, for example, visible in the conducted eccentricity experiments in the Appendix S2.3 & S2.4 (Figs. S4, S5) **[S2:4-8]**.

- *Improved figure caption (Fig. 3, e, f (orig.) / Fig. 4, e, f (rev.)) regarding computation of modulation index, origin of data samples and reference to specific part of the methods section:*
  We improved the figure caption and now clearly state from where data samples for computation of the modulation index distribution are acquired and what served as the baseline activity for calculating the modulation index (Fig. 4) **[10][11]**. Additionally, we extended the methods (Sect. 4.2.2) for a clear description of respective sample acquisition **[45-46][50-51]**.

- *Better explanation of the neurons/step measure (original manuscript, p.11):*
  The caption of Fig. 6 was updated to incorporate a short and intuitive description of the meaning of the neurons/step measure **[13][14]**.

- *Axes labels for Fig. 5, a (orig.) / Fig. 6, a (rev.):*
  We added the missing axes labels to the respective subfigure about V1 activity over time (Fig. 5, a (orig.) / Fig. 6, a (rev.)) **[13][14]**.

- *Unclear role of the cortico-cortical ablation experiment (feedback from V4 to V2) and how it fosters understanding of the growth-cone model beyond known theoretical results from earlier work:*
  We further clarified the intended role the ablation studies play within the investigation Sect. 2.2.3 **[12-15][13-16]**. On the one hand, they serve to verify that the model mechanism is conforming with evidence about the growth-cone hypothesis, and that it indeed working as it was designed. This means that the mechanism's individual components and their putative computational roles and causal relationships work as laid out in the complexity considerations

(Sect. 2.3). On the other hand, they provide the basis for forming model predictions. For example, it provides a prediction about the causal role of the interfacing module in the incremental binding computation and how one could experimentally test this prediction in case the interfacing module would be associated with higher-order thalamic regions.

- *Complexity considerations (rationale behind using a graph-based description, testable predictions from the complexity considerations, mentioning of how complexity results were incorporated into model simulations, further elaboration caption of Fig. 7 (orig.) / Fig. 10 (rev.)):*
  We further motivated the usage of a graph-based description for the conducted complexity analysis. A brief mentioning that the elements of a graph, i.e., nodes and edges, provide a good fit to reason about computation is now prominently placed as the introduction to the caption of Fig. 7 (orig.) / Fig. 10 (rev.) **[22][23]**.

- *Better coverage of previous work during the introduction:*
  The introduction section now covers previous work more extensively and provides a structured overview of the experimental evidence (Sect. 1) **[6][6]**.

- *Inexact and/or confusing writing:*
  We apologize for any confusion that was caused by inexact usage of terminology. Together with improvements to English language (typos and grammar), we also updated many descriptions to make more accurate usage of terminology throughout the paper.

- *Better communication of the relevance of Marr's three levels for the presented work:*
  An important aspect of the presented work is indeed the proposal of a model that explains properties of incremental contour grouping on different levels of description. We followed your suggestion and now present the reader a figure that links different elements of the work to different levels according to Marr (Fig. 2) **[5][5]**.

- *Improved referencing of subfigures and methods subsections for descriptions in the results section:*
  Among other changes to improve clarity and accessibility of the presented work, we incorporated your suggestion to provide more specific references throughout the manuscript.

Response to reviewer 3

We thank you for the fitting – and inspiring – summary of our work and the careful arrangement of the detailed feedback. The major and minor suggestions helped to improve our work regarding clarity and depth. Additionally, we would like to thank you for closely following our methods description to spot any potentially missing information for replication of our work.

Regarding 1. More intuitive illustration of computational mechanisms and operation of the model

- *Illustration of the contour tracing and the growth-cone model (Fig. 1):*
  We improved the illustration of the basic growth-cone mechanism to make it more accessible to the reader (Fig. 1) **[4][4]**. Based on your suggestion we transformed the cases of (no) interference into a sequence of temporal processing of the input stimulus. We further improved the illustration to highlight the important elements for the respective current step of processing. To this end, we made use of gray color and missing distractor receptive field depiction to denote elements that only were relevant during the previous timestep. Further, we highlighted the interference on the top-most scale by using special visual symbols (lightning symbol and hatched interference are). Therefore, changes in target-distractor distance should now be more easily detectable across all panels of the illustration and not only at the bottom row of input panels.

Regarding 2. Discussion of challenges in scaling and calibrating the model to natural continuous curves

- *Generalization of the model mechanism to cases of continuous curves as employed in neurophysiological and behavioral experiments:*
  We extended the simulations to incorporate more complex stimuli, such as curves without abrupt changes among line elements (Fig. 9) **[19][20]**. As can be seen from these results, the model is able to considerably generalize to other stimuli beyond the more simplistic straight lines on which we base the more in-depth results. Importantly, we note that all of these simulations have been conducted with the same model parameters (only stimulus size and value range were adjusted between experiments) and that these model parameters are the ones reported already in the initial submission. Since none of the simulations existed back then, these additional simulations constitute real generalization results. Furthermore, we provide a more thorough discussion covering possible extension of the model Sects. 3.5.1 & 3.5.2 **[37-38][40-41]**.

- *Discussion of fail cases, such as spill-over of attention to nearby distractor curves:*
  Based on the suggestions from you and other reviewers we extended our model simulations for more complex stimuli (Fig. 9) **[19][20]**. This setting motivates a later discussion of limitations, fail cases, and potential extensions of the presented model Sects. 3.5.1 & 3.5.2 **[37-38][40-41]**. Additional results in the Appendix S2.2 & S2.3 highlight successes and failures on likewise demanding configurations **[S2:1-8]**. By this overview, the reader should now get a

clear understanding of what is possible given the presented model and where are its limitations.

- *Elaboration on roles of surround suppression and excitation-inhibition balance:*
  The description of model details now provides insights into how different model parameters and interactions are influencing proper model functioning S1.2 **[S1:2-4]**. As part of this description, we now discuss the role and importance of the balance between excitation and inhibition in the model neurons' local processing. Further insights about the role of surround suppression are now provided for the reader via additional figures that detail the feedback interaction between different orientation channels (Fig. 8) **[17][18]** the selectivity of individual feedback filters (Fig. 12 & Fig. S1) **[41;S1:5][44]**.

Regarding 3. Discussion of challenges in scaling the model to computer vision tasks on natural images

- *Challenges in extending the model for application to natural images and computer vision tasks:*
  We added a more extensive discussion of the challenges when extending the model natural images Sects. 3.5.1 & 3.5.2 **[37-38][40-41]**. This discussion is supported by additional simulations that we have performed given the comments from you, as well as other reviewers (Fig. 9) **[19][20]**. Based on these insights we propose that the main mechanism by how the model computes incremental binding will carry over to natural stimuli, but that the representation needs to be more complex, e.g., containing neurons tuned towards curvature, junctions, or line-ends Sect. 3.5.1 **[38][40]**. We share the impression from your comment, that such representation likely needs to be learned, e.g., by employing the backpropagation algorithm. This is likewise mentioned in the respective discussion Sect. 3.5.3 **[38-39][41-42]**.

- *Detailed discussion of relation to Linsley et al. (NeurIPS, 2018) and whether the proposed model would suggest an alternative artificial neural network architecture:*
  The work of Linsley et al. (NeurIPS, 2018) about the h-GRU model and the pathfinder task now are discussed in greater detail. The discussion is embedded into the broader discussion of how the model generalizes to computer vision tasks Sect. 3.5.3 **[38-39][41-42]**. On the one hand, we specifically discuss commonalities and differences and show how our proposal would provide further understanding of how incremental binding can be implemented mechanistically. By the different impact on cell output between driving feedforward input and modulating contextual recurrent inputs there is a clear distinction between the base representation and incremental grouping computation in our model. On the other hand, we note that the complexity encountered in real-world images likely requires learning of the model's representation, which is something that has been shown for the h-GRU model but is left as future work for the model presented here.

Regarding further comments on the manuscript

- *Ensuring replicability of the presented work by providing all details on methods and technical aspects:*

- We extended the presentation of our methods and now provide all details necessary to ensure replicability. Additional depictions visualize important aspects of connectivity, such as spatial layout of kernels and relative kernel sizes (Fig. 12 & Fig. S1) **[41;S1:5][44]**.

    - *Size of feature maps:*
      We also provide further details about the stimulus sizes (Tab. S2) **[S1:6]** and stimulus creation in general (Appendix S1.3) **[S1:4-7]**. To better account for this increased amount in provided information the existing details on model parameters were rearranged and moved to a new Appendix S1.2) **[S1:2-4]**. There, we further discuss how the sizes of the feature maps are directly derived from the input sizes, since the model architecture is fully convolutional **[S1:4]**.
    - *Outcome for example input, whether contextual interactions appear like association fields, and excitatory-inhibitory connectivity as function of spatial locations and orientations of source and target unit:*
      The simulation results section was extended for an additional figure, which depicts exemplary maps of apical neuron potentials in terms of aggregated potentials and for individual orientation channels (Fig. 8, c) **[17][18]**. The figure helps not only providing further information about the properties of the important apical contextual information (Fig. 8, c), but also to help explain how excitation and inhibition interact locally to solve the incremental binding problem under top-down task-dependent bias (for horizontal versus vertical tracing across the intersection stimulus; Fig. 8, a, b).

- *Discussion of relation to results from Chen et al. (Neuron, 2014):*
  We now discuss the relation to grouping processes developing across multiple visual areas in parallel (Sect. 3.1.2) **[29][31]**. There, the work of Chen et al. (Neuron, 2014) is mentioned as well and embedded within a broader discussion.

- *English language:*
  We greatly improved the presentation of our work in writing and visual communication. We apologize for any unnecessary typos or grammatical errors. The examples mentioned in your comment were corrected, as well as many further smaller and bigger language mistakes. A spellchecking and grammar software has been employed to spot further mistakes. We hope the presentation of the work is now more accessible and understandable.
